# Demographics and histopathological characteristics of palatal lesions: a 24-year retrospective study in an Iranian population

**DOI:** 10.1007/s44445-026-00138-y

**Published:** 2026-05-22

**Authors:** Armin Khaleghi, Yasmin Alimardani, Nafiseh Shamloo

**Affiliations:** 1 Dental Research Center, Shahid Beheshti University of Medical Sciences, Tehran, Iran, Islamic Republic of; 2 Department of Periodontics, Alborz University of Medical Sciences, Karaj, Iran, Islamic Republic of; 3 Department of Oral and Maxillofacial Pathology, Shahid Beheshti University of Medical Sciences, Tehran, Iran, Islamic Republic of

**Keywords:** Palatal neoplasms, Salivary gland neoplasms, Soft tissue neoplasms, Pleomorphic adenoma, Squamous cell carcinoma, Retrospective studies

## Abstract

The palate is a site with a complex anatomy that can develop a variety of lesions of epithelial, salivary gland, and mesenchymal origins. Despite its clinical importance, it has rarely been studied as an independent site. The purpose of this study was to investigate the demographic and pathological features of palatal lesions over 24-years in an Iranian population. A retrospective study was performed on 283 palatal biopsies recorded between 2001 and 2024 at the Department of Oral and Maxillofacial Pathology, Shahid Beheshti Dental School, Tehran, Iran. Lesions were classified into eight histopathological categories. Demographic variables, clinical characteristics, and histological diagnoses were analyzed in RStudio (2024.12.1) using parametric and non-parametric tests with post-hoc adjustment, and significance was set at *p* < 0.05. Among 8,803 total biopsies, 283 cases (3.2%) involved the palate. Epithelial lesions were the largest group (30%), followed by salivary gland lesions (28.3%) and mesenchymal lesions (26.2%). Pleomorphic adenoma was the most common single diagnosis (14.8%). Malignant lesions accounted for 26.5% of the sample, with squamous cell carcinoma being the predominant malignancy. Malignant epithelial lesions occurred at a significantly older age and presented with larger clinical size compared with benign lesions, whereas benign and malignant salivary gland lesions showed overlapping clinical features. Most lesions were located in the posterior palate, a region that is frequently overlooked during routine clinical examination. The palate can be affected by a wide spectrum of lesions with a substantial proportion of malignancies. Older patient age and increased lesion size represent important clinical warnings, particularly for epithelial lesions. Given the limited clinical distinction between benign and malignant salivary gland lesions, biopsy of palatal lesions is strongly recommended regardless of appearance. These findings emphasize the importance of palatal examination and early histopathologic assessment to improve diagnostic accuracy and prognosis.

**Supplementary Information:**

The online version contains supplementary material available at 10.1007/s44445-026-00138-y.

## Introduction

The palate represents a key anatomical structure of the oral cavity, separating it from the nasal compartment and playing an important role in various functions such as mastication, phonation, and swallowing. Anatomically, it is composed of two distinct regions. The anterior two-thirds of the palate is known as the hard palate, and the posterior one-third of the palate is known as the soft palate. The hard palate is lined by a dense, keratinized masticatory mucosa, which is closely related to the underlying bone, whereas the soft palate is covered by flexible non-keratinized mucosa. These structural and histological variations contribute to differences in the spectrum and biological behavior of lesions affecting each region. Notably, the posterior palate contains a greater concentration of minor salivary glands, which may predispose this area to salivary gland pathology that may exhibit subtle or delayed clinical presentation (Neville et al. 2023; Chatterjee 2020; Sharma et al. 2016; Kato et al. 2014; von Arx et al. 2016; Dursun et al. 2018).

Given its anatomical and histological diversity, a wide range of pathological entities may affect the palatal mucosa, including inflammatory conditions, reactive proliferations, benign and malignant neoplasms, immune-mediated disorders, and infections (Abu Rass et al. 2018; Gambhir and Deo 2023). Early recognition and accurate classification of palatal lesions are essential for appropriate management and prognosis, particularly in malignant cases where delayed diagnosis may result in significant morbidity (Zhu et al. 2022).

Most published studies have evaluated palatal lesions as part of broader oral cavity analyses, with limited emphasis on demographic and histopathologic patterns of this specific site (Gupta and Gupta 2013; Caldeira et al. 2012). Furthermore, the lack of a universally accepted classification system for palatal lesions makes comparison between studies difficult. Retrospective analyses based on histopathologic diagnosis remain valuable for identifying patterns of lesion distribution and potential clinical risk indicators.

Therefore, the present study aimed to examine the demographic features, anatomical distribution, and histopathological spectrum of palatal lesions among the Iranian population over a period of 24 years. Through the application of a structured histopathological classification system and excluding lesions with primary bone involvement, the current study hopes to provide clinically useful information that can assist in the early detection and diagnostic decision-making of palatal lesions.

## Materials and methods

### Study design

This retrospective research evaluated oral biopsies of palatal soft-tissue lesions gathered from the archives of the Oral and Maxillofacial Pathology Department, Shahid Beheshti Dental School, Tehran, Iran, between 2001 and 2024, spanning 24-years. To enhance the accuracy of the data, lesions with incomplete or uncertain diagnoses, duplicate records, and lesions that went beyond the palatal mucosa were not included.

### Histopathological classification and data collection

Lesions were classified based on histopathologic criteria according to oral and maxillofacial pathology references rather than a purely clinical grouping. These categories included epithelial lesions, mesenchymal lesions, salivary gland lesions, inflammatory/reactive lesions, hematolymphoid lesions, mucocutaneous lesions, infectious lesions, and non-odontogenic cysts. This classification approach was selected to facilitate site-specific epidemiologic comparison across lesion types rather than neoplastic entities, which is the main focus in WHO categories.

Inflammatory/reactive lesions were defined as entities in which inflammation or reactive tissue response represented the primary histopathologic feature. Lesions characterized by true connective tissue proliferation were classified as mesenchymal, even when reactive in etiology. Odontogenic and primary osseous lesions, as well as cases with underlying bone involvement, were excluded to allow focused evaluation of soft-tissue lesions of the palatal mucosa. In biopsy-based studies, the exact origin of lesions involving bone may be ambiguous due to secondary mucosal changes or limited sampling. Their exclusion minimized diagnostic uncertainty and preserved methodological consistency.

Information on age, sex, size, malignancy, location, clinical presentation, and histological diagnosis was retrieved for analysis. For anatomical localization, palatal lesions were categorized as involving the anterior two-thirds (hard palate) or posterior one-third (soft palate).

### Statistical analysis

Statistical analyses were performed using RStudio (version 2024.12.1). Lesion characteristics were summarized using descriptive statistics according to age, sex, and clinical features, with continuous variables reported as mean and standard deviation. Data distribution was evaluated using the Shapiro–Wilk test, and variance homogeneity was assessed with Levene’s test.

Comparisons among groups for continuous variables were conducted using one-way analysis of variance (ANOVA) followed by Tukey’s honestly significant difference test for normally distributed data, or the Kruskal–Wallis test with Dunn’s post hoc comparisons and Benjamini–Hochberg adjustment when normality assumptions were not met. For two-group comparisons, independent samples t-tests or Mann–Whitney U tests were applied as appropriate. Associations between categorical variables were analyzed using the chi-square test or Fisher’s exact test. A p-value of less than 0.05 was considered statistically significant. Bonferroni correction was applied to adjust for multiple comparisons in analyses of gender distribution across lesion groups. Different correction methods were applied depending on the statistical model to control type I error across multiple comparisons appropriately.

## Results

Out of 8803 lesions that were recorded during this period, 283 cases (3.2%) were located on the palate. Lesions were categorized into eight histopathological groups, with epithelial lesions being the most prevalent (85 cases, 30%), followed by salivary gland lesions (80 cases, 28.3%) and mesenchymal lesions (74 cases, 26.2%). Table 1 summarizes the distribution and demographics of groups. The most common individual lesion was pleomorphic adenoma, accounting for 14.8% of all cases. Supplemental Table 1 shows the demographics of palatal lesions found in our study.

**Table 1:** Demographic and clinical characteristics of palatal lesions by histopathological group

Groups	Frequency N (%)	Sex (m:f)	Age (mean ± SD)	Size in cm (mean ± SD)
Epithelial lesions	85 (30.0%)	36:49	54.11 ± 20.03	1.59 ± 1.40
Salivary gland lesions	80 (28.3%)	41:39	44.71 ± 16.34	2.08 ± 1.32
Mesenchymal lesions	74 (26.2%)	26:48	43.90 ± 19.25	1.49 ± 1.19
Non-odontogenic cysts	17 (6.0%)	13:4	45.12 ± 17.13	1.84 ± 1.13
Mucocutaneous lesions	13 (4.6%)	4:9	51.92 ± 18.67	0.86 ± 0.31
Inflammatory/reactive lesions	10 (3.5%)	5:5	37.40 ± 20.45	1.00 ± 0.54
Hematolymphoid lesions	2 (0.7%)	1:1	68.50 ± 7.78	3.25 ± 2.47
Infectious lesions	2 (0.7%)	2:0	44.50 ± 17.67	-
Total	283 (100%)	128:155	47.53 ± 19.03	1.67 ± 1.29

### Sex

Among 283 cases, 128 (45.2%) occurred in males and 155 (54.8%) in females. Fisher’s Exact Test for sex comparison across all groups yielded a p-value of 0.029, indicating a statistically significant difference in sex distribution. A significant sex difference was found in the non-odontogenic group (*p* = 0.0156), while no significant differences were observed in the other groups.

### Age

**Fig. 1:**
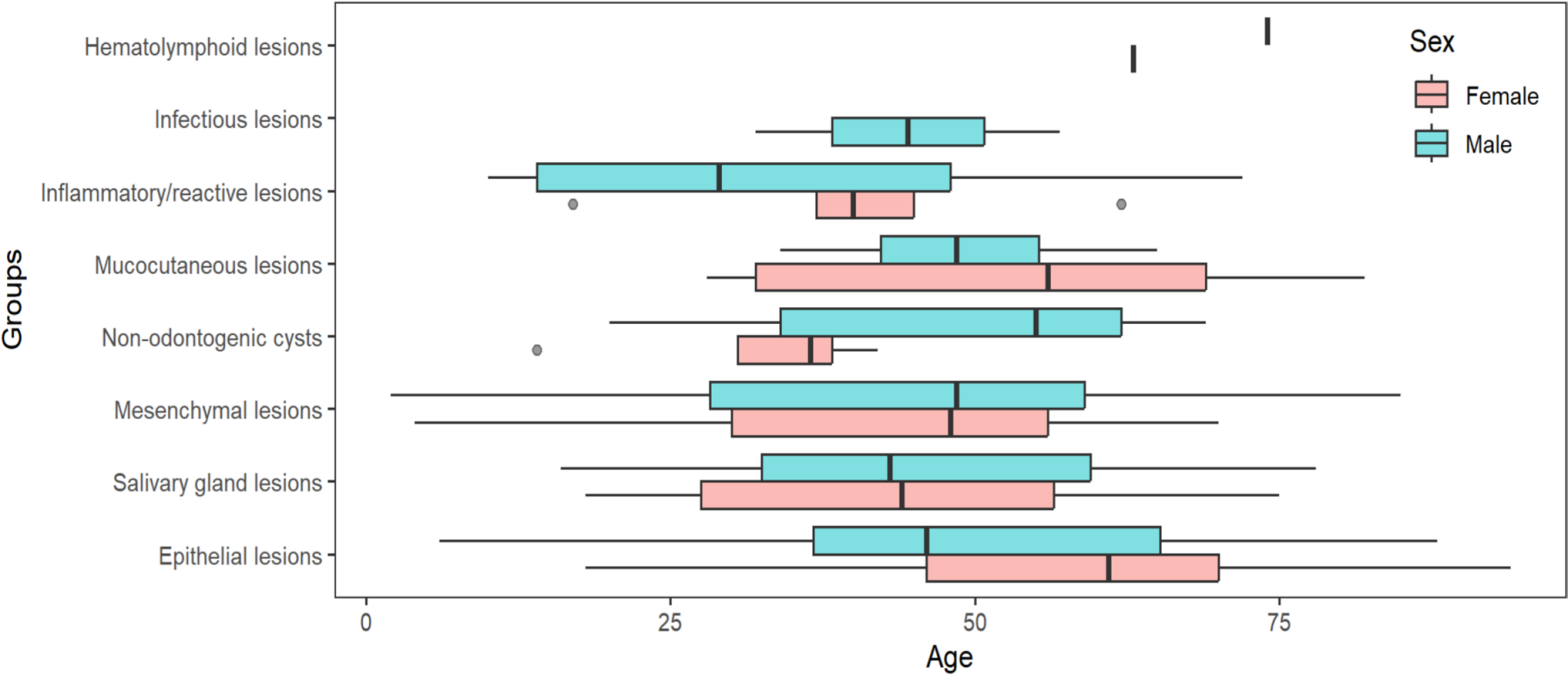
Age distribution of palatal lesions by sex and histopathological group

The age of patients ranged from 2 to 94 years, with a mean age of 47.5 ± 19.0 years (males: 46.9 ± 18.9 years, females: 48.1 ± 19.2 years). The highest prevalence of lesions was in the 50–59 age group (50 cases, 18.3%). Figure 1 illustrates the age distribution of palatal lesions across different histopathological groups, separated by sex. Kruskal–Wallis analysis showed a significant difference in age distribution across groups (*p* = 0.0075). Post-hoc analysis (Dunn’s test with BH correction) revealed that the epithelial group differed significantly from both the mesenchymal lesion (p.adj = 0.0182) and salivary gland lesions (p.adj = 0.0182), while other comparisons were not statistically significant. No significant age differences were found between genders within each histopathological class. The infectious and hematolymphoid groups were excluded from the age-sex analysis due to insufficient data.

### Malignancy

Among all cases, 75 (26.5%) were classified as malignant, with 48 cases (55.2%) reported in females. The mean age for benign and malignant lesions was 43.6 ± 17.7 and 56.72 ± 18.9 respectively, which showed a significant difference between these groups (*p* < 0.001). The most common malignant lesion was squamous cell carcinoma (SCC), comprising 42.6% of all malignancies. Epithelial hyperplasia with dysplasia was the only dysplastic lesion identified on the palate (12 cases, 4.2%), with 75% graded as mild dysplasia and 25% as moderate dysplasia.

**Fig. 2:**
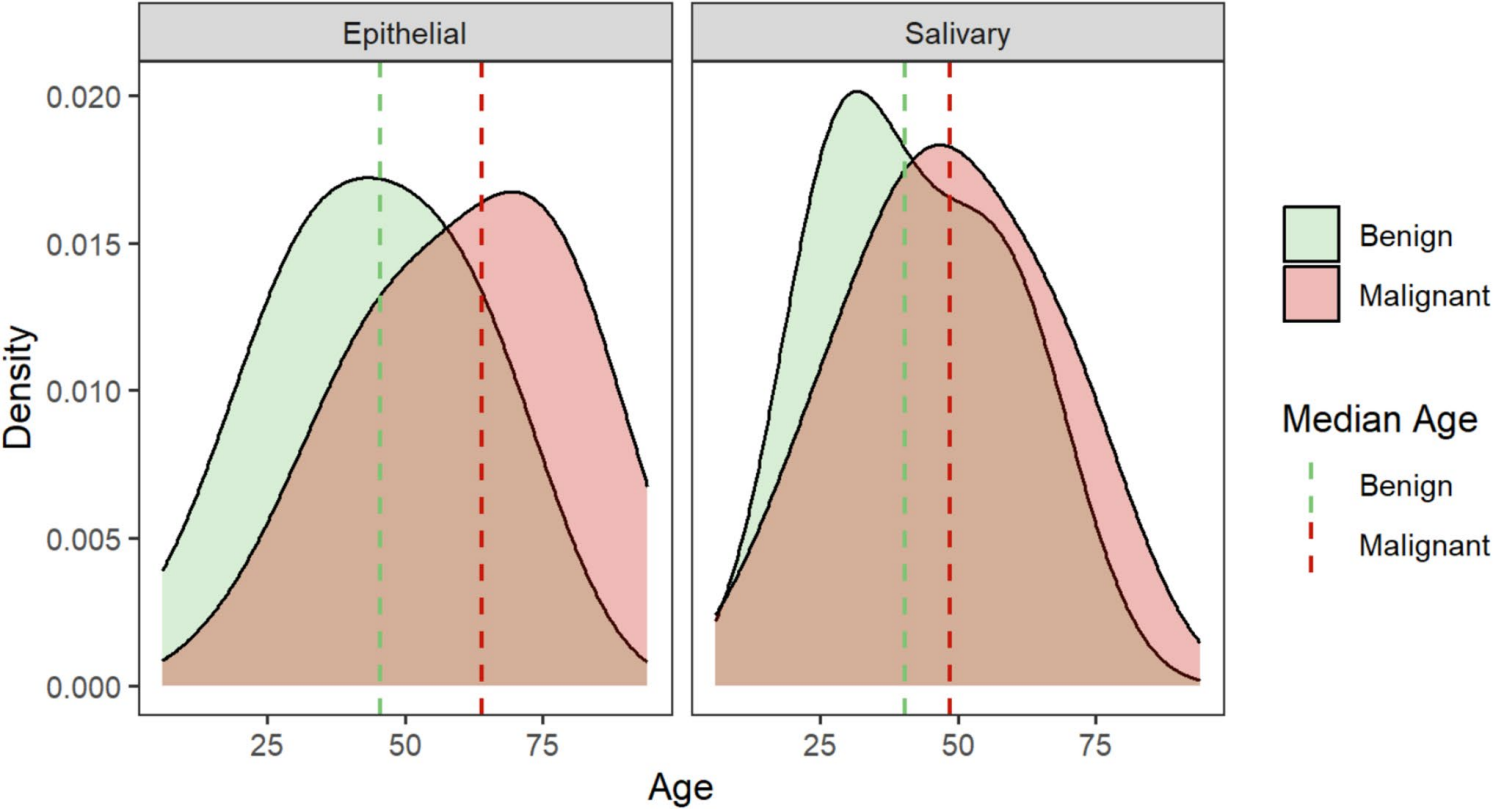
Age distribution in epithelial and salivary gland lesions based on malignancy

**Fig. 3:**
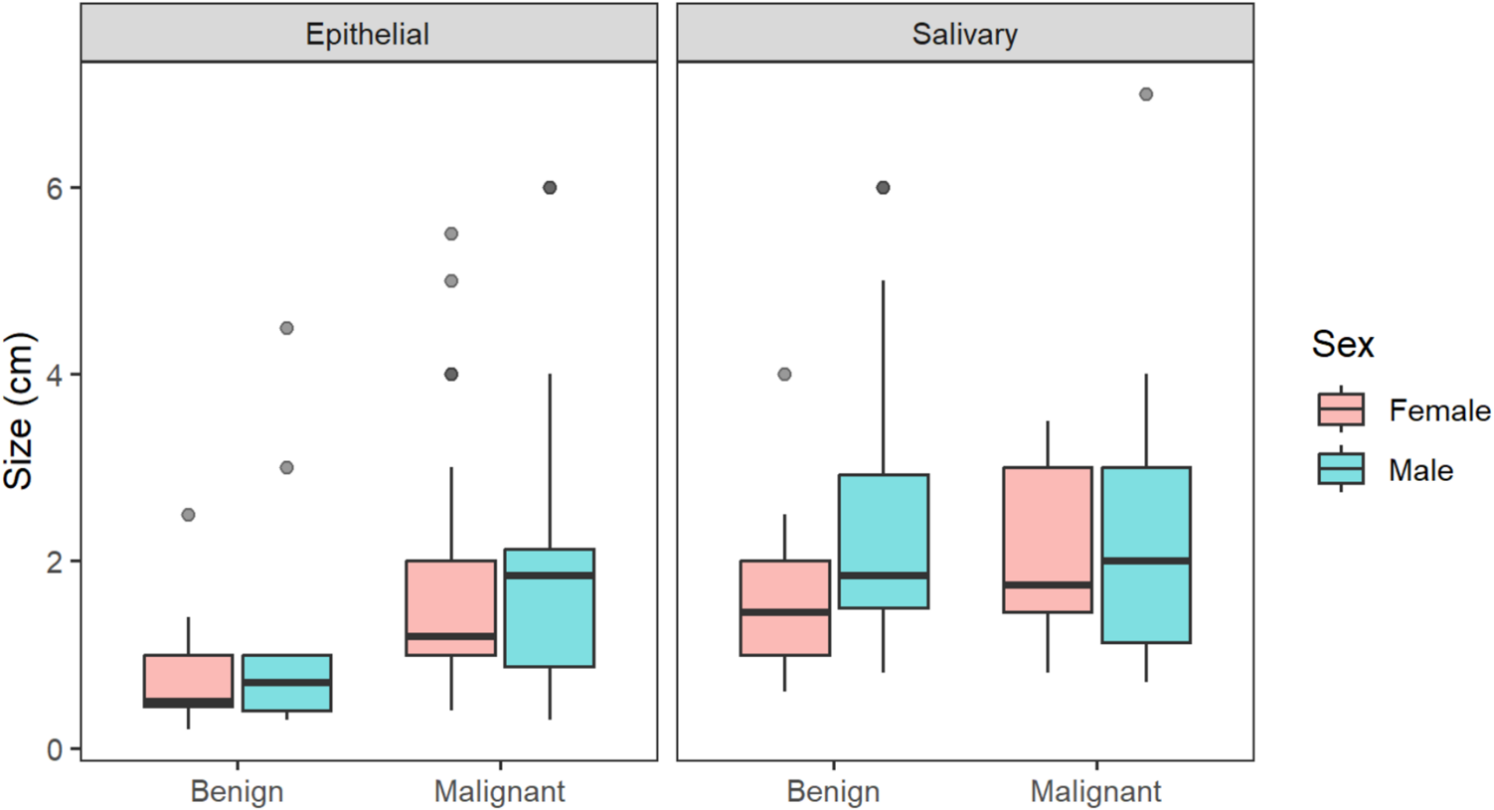
Size comparison in epithelial and salicary gland lesions based on malignancy and sex

Except for a single malignant fibrous histiocytoma in the mesenchymal group and two malignant hematolymphoid cases, all malignant lesions belonged to the epithelial or salivary groups. In these groups, malignancies comprised 63.5% and 37.5% of the lesions. Due to this distribution, further analysis of malignancies focused on these two groups. Figures 2 and 3 illustrate the distribution of age, sex and size in benign and malignant cases within these groups.

For epithelial lesions, malignant cases were significantly older than benign cases (60.9 ± 18.8 vs. 43.4 ± 17.3 years, *p* < 0.001) and had larger tumor sizes (1.87 ± 1.46 cm vs. 0.99 ± 1.05 cm, *p* = 0.014). However, in salivary gland lesions, there was no significant age difference between malignant (48.5 ± 17.1 years) and benign cases (42.4 ± 15.6 years, *p* = 0.108) or in tumor size (2.28 ± 1.48 cm vs. 1.99 ± 1.25 cm, *p* = 0.405).

### Clinical presentation

Lesions were predominantly located in the hard palate (89.0%), while 11% affected the soft palate. Within the hard palate, lesion showed a posterior predilection (78.6%). In terms of consistency, 82.8% of lesions were reported as firm, followed by soft (14.9%), and hard (2.2%). Additionally, 11.3% of lesions presented with ulceration, and 7.8% showed bleeding.

## Discussion

This 24-year retrospective study presents a detailed analysis of the demographic and histopathologic characteristics of palatal lesions diagnosed at Shahid Beheshti University of Dentistry, a major referral center for oral pathology in Tehran, Iran. By isolating the palate as a distinct anatomical region and utilizing a modified version of the Neville classification system (Neville et al. 2023; Shamloo et al. 2024), this investigation offers a focused perspective on a site often underrepresented in epidemiological literature. While prior research has addressed oral lesions more broadly or targeted specific lesion types or age groups (Shamloo et al. 2024; Aydil et al. 2014; Düzlü et al. 2016; Aly et al. 2022), few studies have examined palatal lesions solely. Given the structural complexity and functional significance of the palate, such targeted analysis is both warranted and necessary. Additionally, the site-specific distribution of lesions may reflect local, environmental, etiological, or cultural influences, underscoring the value of regionally derived data (Aly et al. 2022).

### Sex

Previous investigations have reported a male predominance among palatal malignancies (Aydil et al. 2014; Düzlü et al. 2016). In contrast, the present study demonstrated a higher proportion of female patients with palatal malignancies, yielding a male-to-female ratio of 1:1.23. Regarding mesenchymal lesions, a marked female predominance (64.9%) was observed, consistent with previous findings in an Iranian population. Such discrepancies may reflect differences in genetic background, lifestyle factors, healthcare access, or referral patterns, and emphasize the value of studies focused on specific anatomical sites.

### Age

In the present study, palatal malignancies were most frequently diagnosed in older individuals, with a mean age of 56.72 years, consistent with previous reported age trends (Aydil et al. 2014; Düzlü et al. 2016). Mesenchymal lesions occurred at a younger mean age (43.9 years), closely aligning with findings from an Iranian cohort (Shahsavari et al. 2012). Salivary gland lesions demonstrated a similar intermediate age distribution (44.7 years). Overall, these findings suggest that age-related patterns of palatal lesions are broadly comparable across populations, while still reflecting regional variation.

### Malignancy

Malignant lesions accounted for 26.5% of all palatal lesions in the present series, reinforcing the concept of the palate as a relatively high-risk intraoral site. SCC was the most frequent malignancy, in agreement with previous reports identifying SCC as the predominant oral malignancy in Iranian populations (Shamloo et al. 2016). In contrast, other studies reported minor salivary gland tumors as the most common malignancy of the hard palate (Düzlü et al. 2016). In the current study, malignant lesions comprised 37.5% of salivary gland lesions. Despite variations across studies (Beckhardt et al. 1995; Ramesh et al. 2014; Jaafari-Ashkavandi et al. 2011), the substantial proportion of malignant lesions observed here underscores the importance of systematic palatal inspection during routine dental and medical examinations, especially in older patients.

A notable finding of this study is the significant association between epithelial malignancies and increased patient age and lesion size. These variables may serve as valuable clinical indicators of malignancy and should prompt early biopsy. In contrast, the absence of a significant difference between benign and malignant salivary gland lesions highlights the challenge of diagnosing salivary gland tumors. Therefore, biopsy is the only reliable option for diagnosing a palatal lesion. Previous studies have similarly demonstrated the predominance of SCC among oral malignancies and its association with advancing age (Ghai and Sharma 2022; Capote-Moreno et al. 2020). In addition, lesion size has been identified as an important risk factor for malignancy, with larger lesions more likely to exhibit malignant transformation (Kierce et al. 2021; Alves et al. 2017).

### Clinical presentation

Most of the palatal lesions were found in the posterior region. This predominance is likely related to the high concentration of minor salivary glands and relatively thin mucosa in this region, which can facilitate submucosal tumor growth before clinical detection. Furthermore, posterior palatal lesions are less readily visible and may remain asymptomatic for prolonged periods. These factors may contribute to delayed diagnosis, particularly in malignant salivary gland tumors, underscoring the need for routine and thorough examination of the posterior palate during clinical assessments.

### Lesions

Among benign salivary gland tumors, pleomorphic adenoma was the most frequently encountered lesion, consistent with previous studies (Shamloo et al. 2016; Ramesh et al. 2014). Adenoid cystic carcinoma and mucoepidermoid carcinoma were the most common malignant salivary gland tumors, in line with earlier findings from Iranian cohorts (Jaafari-Ashkavandi et al. 2011). Regional studies have reported varying distributions in palatal lesions, suggesting geographic and population-based variability.

### Limitations and conclusion

This study has several limitations, including incomplete documentation in a subset of medical records, which restricted the availability of certain clinical variables. Additionally, as data were obtained from a single referral center, the findings may not be fully generalizable to the broader Iranian population. The extended duration of the study period may also have introduced variability related to diagnostic criteria and record-keeping practices over time.

Despite these limitations, this investigation represents one of the first comprehensive analyses of palatal lesions encompassing a broad diagnostic spectrum using a structured histopathologic classification. By providing site-specific epidemiological data, the present study contributes novel insights into the distribution and characteristics of both benign and malignant palatal lesions. Improved understanding of the demographic and histopathologic profiles of palatal lesions may enhance diagnostic accuracy, support earlier detection, and facilitate more appropriate clinical management, ultimately reducing morbidity associated with these conditions.

## Supplementary Information

Below is the link to the electronic supplementary material.

Supplementary file1 (DOCX 19 KB)

## Data Availability

The data that support the findings of this study are available on request from the corresponding author. The data are not publicly available due to privacy or ethical restrictions.

## References

[CR1] Abu Rass NA, Surougi ER, Baheydarah SM, Baroom AH, AL Ghamdi H, AlTuwayjiri HK, AlMansour NN (2018) Neoplasms of the palate: a review. Egypt J Hosp Med 70(8):1393–1400. 10.12816/0044655

[CR2] Alves AM, Correa MB, Silva KDD et al (2017) Demographic and clinical profile of oral squamous cell carcinoma from a service-based population. Braz Dent J 28(3):301–306. 10.1590/0103-6440201601257 29297550 10.1590/0103-6440201601257

[CR3] Aly MM, Abdul-Aziz MAM, Elchaghaby MA (2022) A retrospective analysis of oral and maxillofacial pathological lesions in a group of Egyptian children over 21 years. BMC Oral Health 22(1):2. 10.1186/s12903-021-02037-6 34996437 10.1186/s12903-021-02037-6 PMC8742446

[CR4] Aydil U, Kızıl Y, Bakkal FK, Köybaşıoğlu A, Uslu S (2014) Neoplasms of the hard palate. J Oral Maxillofac Surg 72(3):619–626. 10.1016/j.joms.2013.08.019 24139293 10.1016/j.joms.2013.08.019

[CR5] Beckhardt RN, Weber RS, Zane R et al (1995) Minor salivary gland tumors of the palate: clinical and pathologic correlates of outcome. Laryngoscope 105(11):1155–1160. 10.1288/00005537-199511000-00003 7475867 10.1288/00005537-199511000-00003

[CR6] Caldeira PC, Ribeiro DC, de Almeida OP, Mesquita RA, do Carmo MA (2012) Tumor of the hard palate. Oral Surg Oral Med Oral Pathol Oral Radiol 113(6):722–727. 10.1016/j.oooo.2011.09.017 22668701 10.1016/j.oooo.2011.09.017

[CR7] Capote-Moreno A, Brabyn P, Muñoz-Guerra MF et al (2020) Oral squamous cell carcinoma: epidemiological study and risk factor assessment based on a 39-year series. Int J Oral Maxillofac Surg 49(12):1525–1534. 10.1016/j.ijom.2020.03.009 32360101 10.1016/j.ijom.2020.03.009

[CR8] Chatterjee S (2020) Swellings of the palate: a review. Indian J Forensic Med Toxicol 14(4):9040–9045. 10.37506/ijfmt.v14i4.13148

[CR9] Dursun A, Öztürk K, Albay S (2018) Development of hard and soft palate during the fetal period and hard palate asymmetry. J Craniofac Surg 29(8):2358–2362. 10.1097/SCS.0000000000005016 30320695 10.1097/SCS.0000000000005016

[CR10] Düzlü M, Karamert R, Bakkal FK et al (2016) The demographics and histopathological features of oral cavity cancers in Turkey. Turk J Med Sci 46(6):1672–1676. 10.3906/sag-1510-97 28081307 10.3906/sag-1510-97

[CR11] Gambhir A, Deo JK (2023) Palatal swellings: a proposed classification and a case report of pleomorphic adenoma of minor salivary gland of the palate. J Maxillofac Oral Surg 22(3):538–542. 10.1007/s12663-022-01808-0 37534360 10.1007/s12663-022-01808-0 PMC10390423

[CR12] Ghai S, Sharma Y (2022) Demographic profile of benign and malignant oral tumors in Central India: a retrospective comparative study. Cureus 14(5):e25345. 10.7759/cureus.25345 35761915 10.7759/cureus.25345 PMC9233233

[CR13] Gupta M, Gupta M (2013) Pleomorphic adenoma of the hard palate. BMJ Case Rep 2013:bcr2013008969. 10.1016/j.ejrad.2022.110566 23576659 10.1136/bcr-2013-008969 PMC3645400

[CR14] Jaafari-Ashkavandi Z, Ashraf MJ, Afandak N (2011) A clinico-pathologic study of 82 intraoral minor salivary gland tumors. Iran Red Crescent Med J 13(9):674–677. 10.5812/kowsar.20741804.2244 22737542 10.5812/kowsar.20741804.2244 PMC3372012

[CR15] Kato H, Kanematsu M, Makita H, Kato K, Hatakeyama D, Shibata T, Mizuta K, Aoki M (2014) CT and MR imaging findings of palatal tumors. Eur J Radiol 83(3):e137–e146. 10.1016/j.ejrad.2013.11.028 24377674 10.1016/j.ejrad.2013.11.028

[CR16] Kierce J, Shi Y, Klieb H, Blanas N, Xu W, Magalhaes M (2021) Identification of specific clinical risk factors associated with the malignant transformation of oral epithelial dysplasia. Head Neck 43(11):3552–3561. 10.1002/hed.26851 34472151 10.1002/hed.26851

[CR17] Neville BW, Damm DD, Allen CM, Bouquot I (2023) Oral and maxillofacial pathology. Saunders Elsevier, St. Louis

[CR18] Ramesh M, Krishnan R, Paul G (2014) Intraoral minor salivary gland tumors: a retrospective study from a dental and maxillofacial surgery centre in Salem, Tamil Nadu. J Maxillofac Oral Surg 13(2):104–108. 10.1007/s12663-013-0489-4 24821999 10.1007/s12663-013-0489-4 PMC4016410

[CR19] Shahsavari F, Khourkiaee SS, Ghasemi MS (2012) Epidemiologic study of benign soft tissue tumors of the oral cavity in an Iranian population. J3D 1(1):10–15. 10.18869/acadpub.3dj.1.1.2

[CR20] Shamloo N, Lotfi A, Motazadian HR, Mortazavi H, Baharvand M (2016) Squamous cell carcinoma as the most common lesion of the tongue in Iranians: a 22-year retrospective study. Asian Pac J Cancer Prev 17(3):1415–1419. 10.7314/apjcp.2016.17.3.1415 27039782 10.7314/apjcp.2016.17.3.1415

[CR21] Shamloo N, Alam M, Khaleghi A (2024) Prevalence of histopathologic types of gingival lesions in the Iranian population: a 22-year retrospective study. Clin Exp Dent Res 10(3):e911. 10.1002/cre2.911 38881225 10.1002/cre2.911 PMC11180844

[CR22] Sharma BB, Sharma S, Jha A, Sharma KD, Sharma JD, Sharma CB (2016) Non-odontogenic hard palate cysts with special reference to globulomaxillary cyst. Plast Aesthet Res 3:302–305. 10.20517/2347-9264.2016.36

[CR23] von Arx T, Lozanoff S. Hard and Soft Palate. InClinical Oral Anatomy: A Comprehensive Review for Dental Practitioners and Researchers 2016 Dec 6 (pp. 199–227). Cham: Springer International Publishing

[CR24] Zhu Y, Wang Y, Tao X, Tang W (2022) Utility of apparent diffusion coefficient histogram analysis in differentiating benign and malignant palate lesions. Eur J Radiol 157:110566 36274361 10.1016/j.ejrad.2022.110566

