# Supplementary material for: Demographics and histopathological characteristics of palatal lesions: a 24-year retrospective study in an Iranian population
Source: Saudi Dent J. 2026 May 22;38(6):75. doi: 10.1007/s44445-026-00138-y (PMC13197501; doi:10.1007/s44445-026-00138-y)
Supplement: Supplementary file 1 — Supplementary file1 (DOCX 19 KB) [file 44445_2026_138_MOESM1_ESM.docx]

#### Supplemental Table 1: Summary of palatal lesions - demographics and distribution

| Groups | Lesions | Frequency N (%) | Sex (m:f) | Age (mean ± SD) |
| --- | --- | --- | --- | --- |
| Epithelial lesions | Squamous cell carcinoma | 32 (11.31%) | 13:19 | 65.97 ± 14.61 |
|  | Squamous papilloma | 17 (6.01%) | 6:11 | 44.76 ± 17.53 |
|  | Epithelial dysplasia | 12 (4.24%) | 2:10 | 57.91 ± 20.35 |
|  | Epithelial Hyperplasia | 10 (3.53%) | 6:4 | 42.60 ± 17.23 |
|  | Melanoma | 7 (2.47%) | 4:3 | 47.17 ± 20.03 |
|  | Others^1^ | 7 (2.47%) | 5:2 | 42.72 ± 49.56 |
| Salivary gland lesions | Pleomorphic adenoma | 42 (14.84%) | 22:20 | 40.50 ± 15.81 |
|  | Mucoepidermoid carcinoma | 14 (4.95%) | 6:8 | 43.46 ± 15.81 |
|  | Adenoid cystic carcinoma | 9 (3.18%) | 5:4 | 51.78 ± 15.01 |
|  | Carcinoma ex pleomorphic carcinoma | 3 (1.06%) | 2:1 | 37.33 ± 23.63 |
|  | Polymorphous adenocarcinoma | 3 (1.06%) | 2:1 | 58.00 ± 7.94 |
|  | Others^2^ | 9 (3.18%) | 4:5 | 55.51 ± 10.83 |
| Mesenchymal lesions | Irritation fibroma | 25 (8.83%) | 4:21 | 46.04 ± 16.54 |
|  | Pyogenic granuloma | 19 (6.71%) | 8:11 | 39.78 ± 20.85 |
|  | Neurofibroma | 7 (2.47%) | 3:4 | 41.00 ± 15.56 |
|  | Giant cell fibroma | 5 (1.77%) | 3:2 | 34.00 ± 30.27 |
|  | Inflammatory papillary hyperplasia | 4 (1.41%) | 2:2 | 39.25 ± 19.38 |
|  | Hemangioma | 3 (1.06%) | 0:3 | 46.67 ± 12.66 |
|  | Leaf-like fibroma | 3 (1.06%) | 1:2 | 70.67 ± 3.79 |
|  | Others^3^ | 8 (2.83%) | 5:3 | 47.54 ± 15.67 |
| Non-odontogenic cysts | Nasopalatine duct cyst | 16 (5.65%) | 12:4 | 46.13 ± 17.16 |
|  | Median palatal cyst | 1 (0.35%) | 1:0 | 29 |
| Mucocutaneous lesions | Lichen Planus | 6 (2.12%) | 3:3 | 38.83 ± 8.28 |
|  | Pemphigus vulgaris | 5 (1.77%) | 1:4 | 58.20 ± 18.83 |
|  | Mucous membrane pemphigoid | 2 (0.71%) | 0:2 | 75.50 ± 9.19 |
| Inflammatory  /reactive  lesions | Inflammatory tissue | 4 (1.41%) | 1:3 | 37.50 ± 14.06 |
|  | Non-specific ulcer | 4 (1.41%) | 3:1 | 44.25 ± 27.28 |
|  | Exogenous pigmentation | 1 (0.35%) | 1:0 | 10 |
|  | Foreign body reaction | 1 (0.35%) | 0:1 | 37 |
| Hematolymphoid lesions | Non-Hodgkin’s Lymphoma | 2 (0.71%) | 1:1 | 68.50 ± 7.78 |
| Infectious lesions | Candidiasis | 1 (0.35%) | 1:0 | 57 |
|  | Mucurmycosis | 1 (0.35%) | 1:0 | 32 |
| Total | | 283 (100%) | 128:155 | 47.53 ± 19.03 |
| ^1^ Epithelial lesions with N<3: verrucous carcinoma (N=2), verruca vulgaris, undifferentiated carcinoma, melanotic macule, nevus and blue nevus (N=1).  ^2^ Salivary gland lesions with N<3: mucocele, adenocarcinoma and canalicular adenoma (N=2), salivary duct carcinoma, chronic sialadenitis and basal cell adenoma (N=1).  ^3^ Mesenchymal lesions with N<3: traumatic neuroma and lipoma (N=2), nodular fasciitis, lymphangioma, bony choristoma and malignant fibrous histiocytoma(N=1). | | | | |
